# Antibiotic prophylaxis prescribing habits in oral implant surgery in the Netherlands: a cross-sectional survey

**DOI:** 10.1186/s12903-019-0981-4

**Published:** 2019-12-12

**Authors:** Fabio Rodríguez Sánchez, Iciar Arteagoitia, Carlos Rodríguez Andrés, Josef Bruers

**Affiliations:** 1 Department of Preventive Medicine and Public Health, University of the Basque Country (UPV/EHU), Barrio Sarriena, s/n, Lejona, 48940 Bilbao, Spain; 2 Department of Oral Health Sciences, Periodontology, Catholic University of Leuven & University Hospitals Leuven, Leuven, Belgium; 3 Department of Stomatology, University of the Basque Country (UPV/EHU), Bilbao, Spain; 4 BioCruces Health Research Institute member, Cruces University Hospital, Barakaldo, Spain; 5 Department of Social Dentistry and Behavioral Sciences, Academic Centre for Dentistry Amsterdam (ACTA), University of Amsterdam and Vrije Universiteit, Amsterdam, the Netherlands; 6 Royal Dutch Dental Association (KNMT), Utrecht, the Netherlands

**Keywords:** Antibiotic prophylaxis prescribing habits, Oral implant surgery, Postoperative infection, Bacterial resistance

## Abstract

**Background:**

There seems to be no consensus on the prescription of prophylactic antibiotics in oral implant surgery. The Dutch Association of Oral Implantology (NVOI) guidelines do not include a clear policy on prophylactic antibiotic prescriptions for oral implant surgery among healthy patients. The purpose of the study was to determine whether antibiotic prophylaxis is commonly prescribed in the Netherlands by general dentists, maxillofacial surgeons and oral implantologists in conjunction with oral implant surgery among healthy patients and to assess the type and amount of prophylactic antibiotic prescribed.

**Methods:**

This observational cross-sectional study is based on a web survey. A questionnaire developed in the United States of America was translated and slightly adjusted for use in the Netherlands. It contained predominantly close-ended questions relating to demographics, qualifications, antibiotic type, prescription duration and dosage. An email including an introduction to the study and an individual link to the questionnaire was sent in February 2018 to a sample of 600 general dental practitioners and all 302 specialized dentists (oral implantologists, periodontists and maxillofacial surgeons) recognized by the NVOI. Overall, 902 questionnaires were anonymously sent. Finally, 874 potential participants were reached. Collected data were analyzed through descriptive statistics.

**Results:**

In total, 218 (24.9%) participants responded to the questionnaire, including 45 females (20.8%) and 171 males (79.2%). Overall, 151 (69.9%) regularly placed oral implants. Of them, 79 (52.7%) prescribe antibiotics only in specific situations, 66 (43.7%) regularly, and 5 (3.3%) did not prescribe antibiotics at all. Overall, 83 participants who prescribe antibiotics did so both pre- and postoperatively (57.2%), 47 only preoperatively (32.4%) and 12 exclusively postoperatively (8.3%). A single dose of 2000 mg of amoxicillin orally one hour prior to surgery was the most prescribed preoperative regimen. The most frequently prescribed postoperative regimen was 500 mg of amoxicillin three times daily for five days after surgery. On average, participants prescribe a total of 7018 mg of antibiotics before, during or after oral implant surgery.

**Conclusions:**

Antibiotic prophylaxis in conjunction with oral implant surgery is prescribed in the Netherlands on a large scale, and recommendations based on the last published evidence are frequently not followed.

## Background

Oral implant surgery is a routine treatment to replace lost teeth [1]. Although oral implants are expected to have a high rate of success, implant failures do occur [2].

Implant failures can be classified as early failures or late failures. Early implant failures occur before the prosthetic restoration, and one of their possible causes might be postoperative infection because of bacterial contamination during the implant insertion [3]. For this reason, the use of perioperative antibiotics has been suggested to prevent postoperative infections and oral implant failures [4].

However, the use of prophylactic antibiotics to reduce the incidence of postoperative infections and oral implant failures remains a controversial issue [5, 6]. Some reviews recommended a single dose of 1 g, 2 g or 3 g of amoxicillin preoperatively but found no evidence to support the postoperative use of a prophylactic antibiotic after oral implant surgery among healthy patients [2, 7].

Markedly, there seems to be no consensus among dentists, oral implantologists, periodontists and maxillofacial surgeons over the use of antibiotics to prevent post-implant infections and oral implant failures [8–12].

The inconsistent use of antibiotics has become an important epidemiologic problem due to the development of bacterial resistance and the risk of superinfection [13], resulting in considerable human and economic costs [14]. Other adverse effects, such as secondary infections, interactions with other medications, gastro-intestinal discomfort, toxicity and allergic reactions, should also be considered [7].

The government and healthcare services of the Netherlands are pursuing several strategies against antibiotic resistance, which include encouraging health professionals to comply with strict guidelines when prescribing antibiotics in an effort to reduce inappropriate antibiotic prescriptions by at least 50% in 2019 [15].

In the Netherlands, oral implant surgery is mainly performed by maxillofacial surgeons and oral implantologists, i.e. dentists who completed a three-year master’s qualification. Antibiotics are the most prescribed drugs in dentistry in the Netherlands. National data revealed that 41.0% of all prescriptions written by dentists during 2015 in the Netherlands were for amoxicillin. Metronidazole and clindamycin accounted for 2.5% of all medications prescribed by dentists [16]..

Nevertheless, the guidelines used in the Netherlands for oral implant surgery do not include a clear policy on the use of prophylactic antibiotics among healthy patients. The Dutch Association of Oral Implantology (NVOI), recommending new publications on this topic in the future, proposed amendments to these guidelines [17].

New research assessing the effectiveness of prophylactic antibiotics for oral implant surgery has been performed in recent years [7, 18, 19].

Moreover, several studies focused on antibiotic prescribing habits in conjunction with oral implant surgeries in different countries have been published recently [8–12]. Markedly, no data were available concerning the situation in the Netherlands. Consequently, it is imperative to assess the current situation in the Netherlands and compare it with similar conditions in other countries.

Therefore, the primary aim of this study was to determine whether antibiotic prophylaxis is commonly prescribed in the Netherlands by general dentists, maxillofacial surgeons and oral implantologists in conjunction with oral implant surgery among healthy patients. The secondary aim was to assess the type and amount (expressed in milligrams: mg) of antibiotics prescribed to evaluate whether any consensus has been reached and the current recommendations made by the last published evidence have been followed [2, 7].

## Methods

This observational cross-sectional study is based on a web survey, and it is reported according to the Strengthening the Reporting of Observational Studies in Epidemiology (STROBE) guidelines [20].

The questionnaire, developed by Deeb et al. (2015) [12], was translated and slightly adjusted for use in the Netherlands to collect data concerning the prescription rates of preventive antibiotics among general dental practitioners, maxillofacial surgeons, periodontists and oral implantologists in conjunction with oral implant therapy. The explicit authorization of Deeb and the co-authors was obtained to use their questionnaire. The translated and adjusted questionnaire was evaluated for comprehensibility and logical order by an experienced oral implantologist, who is involved in the training of dentists in the Netherlands. The formulation of the questions was found adequate and valid to assess the intended objectives.

### Setting

The Netherlands is a member state of the European Union with, in 2018, a population of approximately 17.1 million [21]. In January 2018, roughly 8800 dentists were employed in the Netherlands, including about 320 oral implantologists and 80 periodontists. In addition, at that time, there were about 290 practicing maxillofacial surgeons [22].

### Participants

In February 2018, an email was sent to a representative sample of 600 general dental practitioners, randomly selected from the official register of qualified dentists of the Royal Dutch Dental Association (KNMT), and to all 302 oral implantologists, periodontists and maxillofacial surgeons recognized by the NVOI as oral health care providers who place oral implants and whose email addresses were publicly available.

The only eligibility criteria considered for inviting potential participants to the study was inclusion in the NVOI and KNMT lists. The KNMT maintains an updated file of all licensed dentists in the Netherlands, but does not know whether dentists are active in dentistry. For this reason, it was determined the group of all dentists aged 64 years or less with a known domicile and/or work address in the Netherlands because this group was expected to work in dentistry. A sample of 600 dentists from this group, consisting of about 8800 dentists, was drawn with the SPSS SAMPLE procedure. This was performed by a “Third Party” research institute commissioned by the KNMT. This institute specialized in the management and administration of web surveys and offers support in data collection. The email addresses of NVOI members are publicly available on the NVOI website.

Subsequently, the “Third Party” research institute sent the invitation by e-mail to all potential participants and collected the data. These email messages contained an individual link to a web-based questionnaire and a brief introduction to the study objectives. The participants were assured that the research data were collected anonymously, and it was made clear that by answering the questionnaire, participants consented to the use of the data collected by the survey for the study purpose. All efforts were made to protect the participants’ privacy and anonymity as no personal data of the participants (name, surname, address and telephone number) were collected. In addition, no email addresses were stored or saved by the authors so the participants could not be contacted again. For these reasons, this specific study did not require an ethics statement by an institutional review board (ethics committee) before the study began. Two reminder emails were sent to all potential respondents after two and four weeks; after six weeks, the data collection was closed.

The “Third Party” research institute made the collected data available for use in an encrypted manner so that the authors did not have access to any personal information of the participants, including their email addresses.

A total of 28 potential participants could not be reached because of an incorrect email address. Therefore, the final sample consisted of 874 potential participants: 578 general dental practitioners and 296 oral implantologists, periodontists and maxillofacial surgeons.

### Variables

Information was gathered regarding qualifications and work experience, demographic details and most commonly prescribed preventive antibiotic in case of oral implant placement, including duration and dosage. Based on their statements regarding dosage and period of intake, the total milligrams (mg) prescribed per oral health professional and oral implant surgery was calculated.

### Data sources and measurement

Each link found in the email messages directed the user to a questionnaire, which could only be answered once. The questionnaire contained predominantly close-ended questions. Participants were allowed to add other answer options and additional information (Additional file 1).

### Statistical methods

All data were analyzed using International Business Machines Corporation (IBM) Statistical Package for Social Sciences (SPSS) for Windows Version 24 (IBM Corporation, released 2012, Armonk, New York). To begin, by means of descriptive statistics, an overview was compiled of the respondents’ characteristics in terms of age, sex, type of oral health professional and geographical area. After that, the descriptive analysis continued with only those care providers who had indicated that they place oral implants on a regular basis. Subsequently, their habits regarding prescribing antibiotics before, during or after an oral implant placement were assessed. It was investigated whether antibiotic prescribing habits in oral implant surgery were related to personal characteristics of participants and, for those who prescribe antibiotics, prescription regimens used (chi-squared test and ANOVA). Whether the total amount of prescribed antibiotics varied among certain groups of participants was first investigated by means of ANOVA (F-test) and finally, because the data were not normally distributed, analyzed by means of the Kruskal-Wallis test and Mann-Whitney U test.

## Results

### Participants

Table 1 details the different professionals included in the study. Two participants reported that they were not currently working, and they were excluded from the study group.

**Table 1:** Professional specializations of participants^#1^ and current activity in oral implant surgery

professional specialization	Do place oral implants		Do not place oral implants		Overall	
	n	%	n	%	n	%
General dental practitioner (GDP)	11	5.0	59	27.1	70	32.1
GDP & OI	20	9.2			20	9.2
Oral implantologist (OI)	67	30.7			67	30.7
OI & periodontist (OI-PERIO)	9	4.1	1	0.5	10	4.7
Maxillofacial surgeon (MS)	44	20.2	1	0.5	45	20.6
Other oral health professional^#2^			4	1.8	4	1.8
Not working as oral health professional^#3^			2	0.9	2	0.9
Total	151	69.2	67	30.8	218	100

^#1^ multiple situations possible / ^#2^ dentist for orthodontics (3x), maxillofacial prosthodontist /^#3^ the 2 participants who are not working as oral health professional were excluded from the further analysis of the data

### Descriptive data

In total, 171 males (79.2%) and 45 females (20.8%) responded to the questionnaire. The mean age of the participants was 48.6 years (*SD* = 11.1). While 24.1% were 39 years or younger, 20.8% were between 40 and 49 years old, and 55.1% were 50 years of age or older.

Most participants (92.3%) graduated from a dental school in the Netherlands: Amsterdam (36.6%), Nijmegen (23.8%), Groningen (21.7%) and Utrecht (10.2%). Almost half of the participants (46.3%) were settled in the western part of the country, with 26.4% in the southern, 17.6% in the eastern and 9.7% in the northern regions.

### Oral implant placement and prescribing habits

Table 1 depicts that 69.2% of the participants surveyed indicated that they regularly place oral implants. Of these 151 participants currently performing oral implant surgeries, 66 (43.7%) stated that they regularly prescribe prophylactic antibiotics, while a minority (3.3%, *n* = 5) reported they never do so. In addition, 79 participants (52.3%) indicated they prescribe antibiotics only in certain situations. These situations are presented in Table 2.

**Table 2:** Situations in which participants prescribed antibiotics before, after or during oral implant placement^*#1*^

	n	%
Bone grafting	73	93.6
Sinus perforation	33	42.2
Preoperative implant-site infection	29	37.2
Medically compromised patient^#2^	22	28.2
Past of periodontal disease	14	17.9
Smoking habit	13	16.7
Simultaneous placement of more than 1 dental implant	6	7.7
Dentulous patient^#2^	5	6.4
Other situation^#3^	7	9.0
Total^#4^	78	100

^#1^ multiple situations possible / ^#2^ derived from the option ‘Other situation’, as described by participants / ^#3^ sinus lift surgery (2x), post-operative complications (2x), treatment under anaesthesia, specific location of dental implant placement, based on microbiological test / ^#4^ 1 participant did not indicate situation

No statistically significant relationship was found between any of the participants’ characteristics and their prescribing habits. (Table 3).

**Table 3:** Personal characteristics of participants related to antibiotic prescription habits in oral implant surgery

	Never	Some-times	Always	Overall
Female^#1^		13.9%	7.6%	10.7%
Mean (SD) age in years^#2^	60.0 (4.8)	49.6 (10.1)	51.5 (10.8)	50.8 (10.4)
Type of specialization^#3^				
-General dental practitioner	20.0%	5.1%	9.1%	7.4%
-Oral implantologist and/or periodontist	60.0%	62.0%	65.2%	63.3%
-Oral surgeon	20.0%	32.9%	25.7%	29.3%
Graduation in the Netherlands^#4^	100%	94.9%	90.9%	93.3%
Place of settlement (part of the country)^#5^				
-Southern	60.0%	20.3%	21.2%	22.0%
-Western	40.0%	45.6%	54.5%	49.3%
-Eastern		17.6%	18.2%	17.3%
-Northern		16.5%	6.1%	11.4%
n ^#6^	5	79	66	150

^#1^ *p* 0.343 / ^#2^ *p* 0.071 / ^#3^ *p* 0.597 / ^#4^ *p* 0.520 / ^#5^ *p* 0.173 / ^#6^ 1 participant did not indicate prescription habits

SD: Standard deviation

Table 4 reports the starting times and regimens of the antibiotic prescriptions employed by the participants.

**Table 4:** Antibiotic prescribing regimens and starting time of the prescriptions employed by participants

	n	%	n	%
Only pre-operative			47	32.4
-1 h or immediately	43	29.6		
-1 day prior	2	1.4		
-2 days prior	2	1.4		
Pre- and post-operative			83	57.2
-1 h or immediately	60	41.4		
-1 day prior	18	12.4		
-2 days prior	5	3.4		
Only post-operative			12	8.3
Unknown			3	2.1
Total	145	100	145	100

### Preoperative antibiotics: type, dose and dosage

The majority of participants who prescribe preoperative antibiotics when placing oral implants advise their patients to start one hour prior to treatment (75.2%) or immediately prior to treatment (3.1%). All others stated that they advise their patients to start one day (16.3%) or two days (5.4%) prior to treatment.

Most participants who prescribe prophylactic antibiotics one hour or immediately prior to implant placement prescribe 2000 mg of amoxicillin to be taken orally (70.3%). Furthermore, 9.9% indicated they prescribe 3000 mg of amoxicillin, and 9.9% indicated they prescribe 500 mg of amoxicillin, in both instances to be taken orally.

More than half of the participants (53.9%) who start antibiotic prophylaxis one or two days prior to implant surgery prescribe 500 mg of amoxicillin to be taken orally three times a day. In addition, 19.3% prescribe a combination of 500/125 mg amoxicillin/clavulanic acid to be taken three times a day (Table 5).

**Table 5:** Pre-operative antibiotic regimens prescribed before surgery by participants

1 h or immediately prior				
Antibiotic type	Dose (mg)	Administration	n	%
Amoxicillin	2.000	oral	71	70.3
Amoxicillin	500	oral	10	9,9
Amoxicillin	3.000^#1^	oral	10	9.9
Amoxicillin	1.000	oral	2	2.0
Amoxicillin	other^#2^	oral	2	2.0
Amoxicillin	600	oral	1	1.0
Amoxicillin/Clavulanic acid	500 / 125	oral	3	3.0
Amoxicillin/Clavulanic acid	2.000	oral	1	1.0
Clindamycin	600	oral	1	1.0
Total^#3^			101	100
1 or 2 days prior				
Antibiotic type	Dose (mg)	Dosage	n	%
Amoxicillin	500	oral TID	14	53.9
Amoxicillin	400	oral TID	1	3.8
Amoxicillin	500	oral BID	1	3.8
Amoxicillin	other^#4^	oral TID	1	3.8
Amoxicillin/Clavulanic acid	500 / 125	oral TID	5	19.3
Clindamycin	300	oral BID	1	3.8
Clindamycin	300	oral QID	1	3.8
Erythromycin (ethylsuccinate form)	150	oral TID	1	3.8
Other^#5^	500	oral QD	1	3.8
Total^#6^			26	100

QD: once a day, BID: twice a day, TID: 3 times daily, QID: 4 times daily / ^#1^ mentioned spontaneously / ^#2^ varying / ^#3^ 2 participants did not declare the pre-operative regimen prescribed / ^#4^ 375 mg, it concerns an antibiotic treatment and not antibiotic prophylaxis / ^#5^ Zithromax / ^#6^ 1 participant did not declare the pre-operative regimen prescribed

### Postoperative antibiotics: type, dose and dosage

Of the participants who opt to advise patients to start antibiotic prophylaxis postoperatively, 75.1% prescribe 500 mg of amoxicillin to be taken orally one to four times a day for a period varying from one to eight days (Table 6). Furthermore, 15.2% indicated they prescribe a combination of 500/125 mg amoxicillin/clavulanic to be taken three times a day for a period of five or seven days.

**Table 6:** Post-operative antibiotic regimens prescribed after surgery by participants

Antibiotic type	Dose (mg)	Dosage	Duration	n	%
Amoxicillin	250	oral TID	5 days	1	1.1
Amoxicillin	400	oral TID	5 days	1	1.1
Amoxicillin	500	oral QD	3 days	1	1.1
Amoxicillin	500	oral BID	7 days	1	1.1
Amoxicillin	500	oral TID	1 day	4	4.2
Amoxicillin	500	oral TID	3 days	2	2.2
Amoxicillin	500	oral TID	5 days	29	31.4
Amoxicillin	500	oral TID	6 days	1	1.1
Amoxicillin	500	oral TID	7 days	24	26.1
Amoxicillin	500	oral TID	8 days	1	1.1
Amoxicillin	500	oral TID	other^#1^	2	`2.2
Amoxicillin	500	oral QID	2 days	1	1.1
Amoxicillin	500	oral QID	4 days	1	1.1
Amoxicillin	500	oral QID	5 days	2	2.2
Amoxicillin/Clavulanic acid	500/125	oral TID	1 day	1	1.1
Amoxicillin/Clavulanic acid	500/125	oral TID	5 days	6	6.5
Amoxicillin/Clavulanic acid	500/125	oral TID	7 days	8	8.7
Amoxicillin/Clavulanic acid	500/125	oral QID	6 days	1	1.1
Amoxicillin/Clavulanic acid	500/125	oral QID	7 days	1	1.1
Clindamycin	300	oral TID	7 days	1	1.1
Clindamycin	300	oral QID	5 days	1	1.1
Clindamycin	300	oral QID	7 days	1	1.1
Other^#2^	500	oral QD	2 days	1	1.1
Total^#3^				92	100

QD: once a day, BID: twice a day, TID: 3 times daily, QID: 4 times daily / ^#1^ unknown /^#2^ Zithromax / ^#3^ 3 participants did not declare the postoperative regimen prescribed

### Amounts of prescribed antibiotics

On average, participants stated that they prescribe a total of 7018 mg (*SD* = 4325 mg) of prophylactic antibiotics before, during or after oral implant surgery, varying from 500 mg to 14,600 mg with the median lying at 8000 mg. Three participants did not indicate their prescribing regimens, and six did not declare the number of milligrams prescribed (Table 7).

**Table 7:** Total amount of antibiotics (mg) prescribed by participants related to their type of working situation and prescription habits

	Mean	SD	Mean Rank ^#1^	P
Type of oral health professional				0.029
-General dental practitioner (GDP)	4150	3705	39.3	
-Maxillofacial surgeon (MS)	6883	4195	68.3	
-Oral implantologist (OI)	7969	4179	75.8	
Antibiotic prescription habits				0.003
-Sometimes	7799	4173	77.9	
-Always	5913	4059	57.9	
Antibiotic prescription regimen				< 0.0001
-Only pre-operative	2060	463	24.9	
-Only post-operative	9250	1545	84.1	
-Pre- and post-operative	9598	2963	91.8	
Total	7018	4235		
n = 136 ^#2^				

^#1^ Kruskal-Wallis test or Mann-Whitney test / ^#2^ 3 participants did not indicate prescribing regimens and 6 participants did not completely declare the number of mg or did not declare it at all

Notably, maxillofacial surgeons prescribe significantly more antibiotics than oral implantologists and general practitioners (7969 mg versus 6883 mg and 4150 mg; *p* = 0.03).

In particular, the difference between maxillofacial surgeons and general practitioners was statistically significant (*p* = 0.02). Participants who only opted for antibiotics prior to treatment reported prescribing significantly smaller amounts than their colleagues who opted for antibiotics only after treatment and prior to as well as after treatment (2060 mg versus 9250 mg and 9598 mg; *p* < 0.001). Furthermore, it appears that participants who regularly prescribe an antibiotic prophylaxis in conjunction with oral implant surgery prescribe significantly smaller amounts of antibiotics than participants who prescribe prophylactic antibiotics only in certain circumstances. Conversely, participants who prescribe prophylactic antibiotics only in certain circumstances when inserting oral implants indicated they prescribe longer regimens (pre- and postoperatively) than participants who indicated they regularly prescribe prophylactic antibiotics (*p* = 0.04).

## Discussion

### Key results

Considering the latest evidence published on this topic, more than two-thirds of the participants in this study do not follow an adequate prophylactic antibiotic regimen. They prescribe prophylactic antibiotics in many situations not defined by the guidelines proposed by the NVOI [17]. Moreover, there appears to be a lack of consensus regarding the indications for prescribing prophylactic antibiotics in conjunction with oral implant surgery among healthy patients as well as regarding the antibiotic of choice and the regimen selection.

### Limitations

Before the start of the study, it was unclear how many dentists and maxillofacial surgeons insert oral implants in the Netherlands. For that reason, the research group was composed of all maxillofacial surgeons, periodontists and oral implantologists who are recognized to routinely perform oral implant surgery and an additional random sample of general dental practitioners, who are also qualified to perform this treatment. In this way, the chance of any selection bias was minimized.

Overall, the response rate of 24.9% was not high, but it was considered adequate for a web questionnaire [23]. While there was no certainty that all dentists, maxillofacial surgeons, periodontists and oral implantologists placing oral implants in the Netherlands were reached, it was considered that the participants in this study properly represented the target population.

The questionnaire was completely anonymous to encourage respondents to answer the questions as truthfully as possible to avoid risk of bias. Nevertheless, the authenticity of the answers obtained was difficult to control. Moreover, as in most survey studies, it is uncertain whether respondents’ statements about their behavior match their behavior in practice.

### Interpretation

In accordance with Esposito et al. [2], the NVOI guidelines acknowledge that a single preoperative dose of oral antibiotics may slightly decrease implant failures. Nevertheless, standard antibiotic prophylaxis in conjunction with oral implant placement among healthy patients is not recommended. The last published reviews stress the lack of evidence supporting the use of postoperative antibiotics exclusively after surgery or as a combination with preoperative antibiotics [2, 7]. Preoperative antibiotics are only indicated as bacterial endocarditis prophylaxis for patients with orthopedic implants or in implant surgeries performed on infected sites. Following the NVOI guidelines, the first treatment choice in these situations should be a single dose of oral amoxicillin and clavulanic acid (1000/250 mg) one hour before surgery or, in the event of allergies, oral clindamycin (600 mg) one hour prior to treatment [17]. However, the last published evidence recommends a single preoperative dose of 1 g, 2 g or 3 g of amoxicillin [2, 7]. It has also been described recently that penicillin-allergic patients treated with clindamycin may present more risk of suffering oral implant failures [24].

This study confirms a lack of agreement on the prescription of prophylactic antibiotics in oral implant surgery, as already demonstrated in many other medical situations in which this treatment is an option [25]. However, in comparison with their colleagues in other countries, dentists, maxillofacial surgeons, periodontists and oral implantologists in the Netherlands seem to prescribe a smaller range of antibiotic types and regimens and seem more cautious in prescribing prophylactic antibiotics. In the UK, a study revealed that approximately 72% of dentists prescribe antibiotics for all oral implant surgery procedures [9], while a Swedish study revealed that 74% of dentists routinely prescribe antibiotics in conjunction with oral implant surgery [10].

The prescription patterns of maxillofacial surgeons in the USA revealed that 96% prescribe prophylactic antibiotics for healthy patients [12], and similar research in Spain found that almost 90% of the dentists studied regularly prescribe antibiotics among healthy patients [11]. Conversely, it was found that the proportion of dentists in Jordan who prescribe antibiotics in all implant surgeries was around 50% [8].

The present study found differences in the prescription duration between participants who prescribe prophylactic antibiotics systematically (more often opting for short-term regimens) and those professionals who prescribe prophylactic antibiotics only in certain instances (more often opting for long-term regimens). This is probably related to the fact that the latter more often prescribe prophylactic antibiotics for a particular reason after reflection and a decision-making process.

The discrepancies in the average amount of prophylactic antibiotics prescribed by maxillofacial surgeons and general dentists in the Netherlands might be explained by the relative contrast in the complexity of the surgeries executed by each group. Maxillofacial surgeons may face more complicated treatments than general dentists, which could generate the prescription of heavier prophylactic regimens.

Similar to health care professionals worldwide, oral health professionals performing implant surgery in the Netherlands prescribe too often and too many prophylactic antibiotics. This may lead to an alarming risk of bacterial resistance and the development of other adverse reactions to antibiotics, which potentially generates a serious public health problem and consequently, high societal and economic costs.

### Generalisability

This survey was conducted among oral healthcare professionals in the Netherlands registered as general dental practitioners, oral implantologists or maxillofacial surgeons who graduated in representative proportions from various dental schools. Combined with the fact that a substantial proportion of the oral health professionals who perform oral implant surgeries participated in this study, it is plausible that the results of the survey broadly reflect the national situation in the Netherlands.

## Conclusions

Antibiotic prophylaxis in conjunction with oral implant surgery among healthy patients is prescribed in the Netherlands on a large scale by general dentists, maxillofacial surgeons, periodontists and oral implantologists. In addition, recommendations based on the last published evidence are frequently not being followed. For this reason, more attention should be paid to the implementation of existing guidelines. New research assessing the effects of different prophylactic antibiotic types, dosages and regimens in healthy patients is also necessary to update evidence-based guidelines.

## Supplementary information

**Additional file 1.** Questionnaire. This file contains the English version of the survey sent to participants.

## Data Availability

The datasets used and/or analyzed during the current study are available from the corresponding author on reasonable request.

## Abbreviations

GDP: General dental practitioner
IBM: International Business Machines Corporation
KNMT: Royal Dutch Dental Association
Mg: Milligrams
MS: Maxillofacial surgeon
NVOI: Dutch Association of Oral Implantology
OI: Oral implantologist
OI-PERIO: Oral implantologist and periodontist
SPSS: Statistical Package for Social Sciences
STROBE: Strengthening the Reporting of Observational Studies in Epidemiology
