# Supplementary material for: Antibiotic prophylaxis prescribing habits in oral implant surgery in the Netherlands: a cross-sectional survey
Source: BMC Oral Health. 2019 Dec 12;19:281. doi: 10.1186/s12903-019-0981-4 (PMC6909651; doi:10.1186/s12903-019-0981-4)
Supplement: Supplementary file 1 — Additional file 1. Questionnaire. This file contains the English version of the survey sent to participants. [file 12903_2019_981_MOESM1_ESM.pdf]

- Assume that your patients are healthy and do not have antibiotic allergies to answer these questions.
- Choose the answer that is most applicable to your situation / clinical practice.

1	Do you prescribe antibiotics before, during or after placement of dental implants?		
	1	No, never	(to question 10)
	2	Yes, sometimes	(to question 2)
	3	Yes, always	(to question 3)

2	Can you briefly describe the situation(s) in which you prescribe an antibiotic before, during or after placing dental implants?<br>More answers are possible.	
	1	in the case of bone augmentation
	2	if a patient has a parodontal history
	3	if a patient smokes
	4	in the event of pre-operative infection of the implant site
	5	in the case of sinus perforation
	6	if several implants are placed
	7	different situation, namely:

3	When do you prescribe antibiotics?		
	1	Pre-surgery only (preoperative)	(to question 4 / 5, 6 or 7)
	2	Post-surgery only (postoperative)	(to question 8)
	3	both before and after surgery (before and after surgery)	(to question 4 / 5, 6 or 7 / 8)

4	If you prescribe that the patient should start before placing the implants, when should the patient start antibiotic prophylaxis?		
	1	2 days before the surgical procedure	(to question5 / 8 or 10)
	2	1 days before the surgical procedure	(to question5 / 8 or 10)
	3	1 hour before the surgical procedure	(to question6 / 8 or 10)
	4	Immediately prior to the surgical procedure	(to question7 / 8 or 10)

5	*You prescribe that the patient should (also) take antibiotic prophylaxis 1 or 2 days before placing the implants.<br>Could you indicate below what type of antibiotic, at what dosage and what administration you will prescribe?*						
		Type		*Dose*		*Posology*	*Administration*
	1	Amoxicillin	1	150 mg	1	oraal 1 x daags	intra musculair
	2	Amoxicillin/Clavulanic	2	250 mg	2	oraal 2x daags	intraveneus
	3	Clindamycin	3	300 mg	3	oraal 3x daags	other, namely:
	4	Erythromycin (ethyl succinate form)	4	400 mg	4	oraal 4x daags	
	5	Erythromycin (base stearate form)	5	500 mg	5	intra musculair	
	6	Cefalexin	6	500 / 125 mg	6	intraveneus	
	7	Penicillin VK	7	800 mg	7	other, namely:	
	8	other, namely:	8	875 mg			
			9	875 / 125 mg			
			10	1000 mg			
			11	other, namely:			

6	You prescribe that the patient should (also) take antibiotic prophylaxis 1 hour before placing the implants.<br>Please indicate below which type of antibiotic, in which Dose and which Posology you are prescribing					
		Type		*Dose*		*Administration*
	1	Amoxicillin	1	250 mg	1	oraal
	2	Ampicillin	2	500 mg	2	intra musculair
	3	Amoxicillin/Clavulanic	3	500 / 125 mg	3	intraveneus
	4	Cefazolin	4	600 mg	4	other, namely:
	5	Clindamycin	5	800 mg		
	6	Erythromycin (ethyl succinate form)	6	875 mg		
	7	Erythromycin (base stearate form)	7	875 / 125 mg		
	8	Cefalexine	8	1000 mg (1g)		
	9	Penicillin VK	9	1600 mg		
	10	other, namely:	10	2000 mg (2g)		
			11	other, namely:		

7	*You prescribe that the patient should (also) take antibiotic prophylaxis immediately before placing the implants.<br>Could you indicate below what type of antibiotic, at what concentration and what dose you are prescribing?*					
		Type		*Dose*		*Administration*
	1	Amoxicillin	1	250 mg	1	oraal
	2	Ampicillin	2	500 mg	2	intra musculair
	3	Amoxicillin/Clavulanic	3	500 / 125 mg	3	intraveneus
	4	Cefazolin	4	600 mg	4	other, namely:
	5	Clindamycin	5	800 mg		
	6	Erythromycin (ethyl succinate form)	6	875 mg		
	7	Erythromycin (base stearate form)	7	875 / 125 mg		
	8	Cefalexine	8	1000 mg (1g)		
	9	Penicillin VK	9	1600 mg		
	10	other, namely:	10	2000 mg (2g)		
			11	other, namely:		

8	*You prescribe that the patient should (also) take antibiotic prophylaxis after placing the implants. Could you indicate below what type of antibiotic, at what concentration and what dose you are prescribing?*					
		Type		*Dose*		*Posology*
	1	Amoxicillin	1	150 mg	1	oraal 1x daags
	2	Amoxicillin/Clavulanic	2	250 mg	2	oraal 2x daag
	3	Clindamycin	3	300 mg	3	oraal 3x daag
	4	Erythromycin (ethyl succinate form)	4	400 mg	4	oraal 4x daags
	5	Erythromycin (base stearate form)	5	500 mg	5	intra musculair
	6	Cefalexine	6	500 / 125 mg	6	intraveneus
	7	Penicillin VK	7	800 mg	7	other, namely:
	8	other, namely:	8	875 / 125 mg		
			9	1000 mg (1g)		
			10	other, namely:		

9	How many days should the patient take the antibiotic prophylaxis?	
	1	1 day
	2	2 days
	3	3 days
	4	4 days
	5	5 days
	6	6 days
	7	7 days
	8	8 days
	9	9 days
	10	10 days
	11	11 days
	12	12 days
	13	13 days
	14	14 days
	15	15 days
	16	different number of days, namely:

10	What is your gender?	
	1	male
	2	female

11	What's your age?	
	1	30 years or younger
	2	31 - 40 years
	3	41 - 50 years
	4	51 - 60 years
	5	61 years or older

12	At which university did you obtain your dental degree?	
	1	Universiteit van Amsterdam
	2	Vrije Universiteit Amsterdam
	3	Academisch Centrum Tandheelkunde Amsterdam
	4	Rijksuniversiteit Groningen
	5	Rijksuniversiteit Utrecht
	6	Radboud Universiteit Nijmegen
	7	universiteit in buitenland, namelijk:

13	In which province is the hospital, clinic or practice where you are active in oral implantology located?	
	1	Groningen
	2	Friesland
	3	Drenthe
	4	Overijssel
	5	Flevoland
	6	Noord Holland
	7	Zuid Holland
	8	Utrecht
	9	Gelderland
	10	Zeeland
	11	Noord Brabant
	12	Limburg
